# The Redox-Active Manganese(III) Porphyrin, MnTnBuOE-2-PyP^5+^, Impairs the Migration and Invasion of Non-Small Cell Lung Cancer Cells, Either Alone or Combined with Cisplatin

**DOI:** 10.3390/cancers15153814

**Published:** 2023-07-27

**Authors:** Rita B. Soares, Rita Manguinhas, João G. Costa, Nuno Saraiva, Nuno Gil, Rafael Rosell, Sérgio P. Camões, Ines Batinic-Haberle, Ivan Spasojevic, Matilde Castro, Joana P. Miranda, Paula Guedes de Pinho, Ana S. Fernandes, Nuno G. Oliveira

**Affiliations:** 1 Research Institute for Medicines (imed.ULisboa), Faculty of Pharmacy, Universidade de Lisboa, Av. Professor Gama Pinto, 1649-003 Lisboa, Portugal; soares.rita@edu.ulisboa.pt (R.B.S.); rmanguinhas@campus.ul.pt (R.M.); sergiocamoes@campus.ul.pt (S.P.C.); mcastro@ff.ulisboa.pt (M.C.); jmiranda@ff.ulisboa.pt (J.P.M.); 2 Lung Unit, Champalimaud Clinical Centre, Champalimaud Foundation, Av. Brasília, 1400-038 Lisbon, Portugal; nuno.gil@fundacaochampalimaud.pt; 3 Universidade Lusófona’s Research Center for Biosciences & Health Technologies (CBIOS), Campo Grande 376, 1749-024 Lisboa, Portugal; jgcosta@ulusofona.pt (J.G.C.); nuno.saraiva@ulusofona.pt (N.S.); 4 Laboratory of Cellular and Molecular Biology, Institute for Health Science Research Germans Trias I Pujol (IGTP), Campus Can Ruti, Ctra de Can Ruti, Camí de les Escoles, s/n, 08916 Badalona, Barcelona, Spain; rrosell@iconcologia.net; 5 Department of Radiation Oncology, Duke University School of Medicine, Durham, NC 27710, USA; ibatinic@duke.edu; 6 Department of Medicine, Duke University School of Medicine, Durham, NC 27710, USA; ivan.spasojevic@duke.edu; 7 PK/PD Core Laboratory, Duke Cancer Institute, Duke University School of Medicine, Durham, NC 27710, USA; 8 Associate Laboratory i4HB-Institute for Health and Bioeconomy, Department of Biological Sciences, Laboratory of Toxicology, Faculty of Pharmacy, University of Porto, 4050-313 Porto, Portugal; pguedes@ff.up.pt; 9 UCIBIO, REQUIMTE, Laboratory of Toxicology, Faculty of Pharmacy, University of Porto, 4050-313 Porto, Portugal

**Keywords:** SOD mimic, Mn porphyrin, MnTnBuOE-2-PyP^5+^ (BMX-001), non-small cell lung cancer, cisplatin, migration, invasion, cytotoxicity

## Abstract

**Simple Summary:**

Oxidative stress affects several features of cancer, including those related to metastases formation. Non-small cell lung cancer (NSCLC) is commonly detected in advanced stages when metastases have already developed. MnTnBuOE-2-PyP^5+^ (MnBuOE, BMX-001) is a redox-active Mn porphyrin-based drug, and a mimic of superoxide dismutase, that is currently in clinical trials for several types of cancer. Nevertheless, there is still insufficient information regarding the effect of this compound on lung cancer. Here, we aim at filling this gap by assessing the impact of MnBuOE alone or combined with cisplatin—a frequently used platinum-based drug in chemotherapy—on the viability and migration of two NSCLC cell lines. Although MnBuOE only displayed moderate effects on suppressing cancer cell viability, it significantly reduced collective and individual migration and invasion in both types of cells, especially when combined with cisplatin. The data thus support the therapeutic potential of MnBuOE as an anti-metastatic drug for the treatment of NSCLC.

**Abstract:**

Manganese(III) porphyrin MnTnBuOE-2-PyP^5+^ (MnBuOE, BMX-001) is a third-generation redox-active cationic substituted pyridylporphyrin-based drug with a good safety/toxicity profile that has been studied in several types of cancer. It is currently in four phase I/II clinical trials on patients suffering from glioma, head and neck cancer, anal squamous cell carcinoma and multiple brain metastases. There is yet an insufficient understanding of the impact of MnBuOE on lung cancer. Therefore, this study aims to fill this gap by demonstrating the effects of MnBuOE on non-small cell lung cancer (NSCLC) A549 and H1975 cell lines. The cytotoxicity of MnBuOE alone or combined with cisplatin was evaluated by crystal violet (CV) and/or 3-(4,5-dimethylthiazol-2-yl)-5-(3-carboxymethoxyphenyl)-2-(4-sulphophenyl)-2H-Tetrazolium (MTS) reduction assays. Intracellular ROS levels were assessed using two fluorescent probes. Furthermore, the impact of MnBuOE alone or in combination with cisplatin on collective cell migration, individual chemotactic migration and chemoinvasion was assessed using the wound-healing and transwell assays. The expression of genes related to migration and invasion was assessed through RT-qPCR. While MnBuOE alone decreased H1975 cell viability at high concentrations, when combined with cisplatin it markedly reduced the viability of the more invasive H1975 cell line but not of A549 cell line. However, MnBuOE alone significantly decreased the migration of both cell lines. The anti-migratory effect was more pronounced when MnBuOE was combined with cisplatin. Finally, MnBuOE alone or combined with cisplatin significantly reduced cell invasion. MnBuOE alone or combined with cisplatin downregulated *MMP2*, *MMP9*, *VIM*, *EGFR* and *VEGFA* and upregulated *CDH1* in both cell lines. Overall, our data demonstrate the anti-metastatic potential of MnBuOE for the treatment of NSCLC.

## 1. Introduction

Oxidative stress is a common characteristic of several pathologies, including cancer. It affects several cancer features, including complex processes leading to metastasis, angiogenesis and invasiveness [1]. Increased levels of reactive oxygen species (ROS) can modulate oncogenic signaling pathways, mutagenesis and genomic instability in cancer cells, which can promote cancer progression [2]. Importantly, numerous studies have demonstrated that cancer cells possess higher ROS levels than healthy cells, which they can utilize for their own advantage, i.e., survival and proliferation [2,3,4,5]. Importantly, cancer cells also have limited ability to control ROS levels. Therefore, chemotherapy and radiotherapy aim to further increase the levels of reactive species in cancer cells to an extent that promotes their death [6]. Besides having generally higher ROS levels than healthy cells, it has also been demonstrated that cancer cells, in general, may have a lower ability to detoxify hydrogen peroxide (H_2_O_2_) due to lower levels of functional antioxidants than non-malignant cells [7]. Although such conditions are beneficial for the survival and proliferation of cancer cells, there is a delicate balance. At high concentrations, H_2_O_2_ becomes cytotoxic to cancer cells, leading to their death [8]. Redox-directed cancer therapies have been recently developed to further increase H_2_O_2_ concentration and in turn enhance the efficacy of radio- and chemotherapy [9].

Mn porphyrin-based SOD mimics (SODm) have been studied as redox-directed therapeutic strategies for different types of pathologies, e.g., cardiovascular, neurodegeneration, diabetes and cancer [10]. SODm are synthetic compounds with the ability to mimic the properties of native SOD enzymes, dismuting O_2_^•−^ into H_2_O_2_ and oxygen [11]. In addition to interacting with O_2_^•−^, the redox properties of these compounds allow them to undergo diverse reactions with other biomolecules, such as ONOO^−^, H_2_O_2_ and NO_2_ [8]. Previous studies have provided evidence that the major mode of action of these compounds is the H_2_O_2_-driven catalytic oxidation of protein cysteines. SODm could utilize H_2_O_2_, produced by radio- and chemotherapy, as well as H_2_O_2_ produced during their cycling with cellular reductants. This, in turn, would modify the activities of numerous proteins such as those of transcription factors NF-kB and Nrf2 [12,13,14]. The activation of Nrf2 would lead to upregulation of different antioxidants, including MnSOD. Thus, in addition to directly removing O_2_^•−^, Mn porphyrins could also upregulate MnSOD and, therefore, indirectly reduce levels of O_2_^•−^. The differential activity of SODm in normal vs. cancer cells is the consequence of the differences in the levels of H_2_O_2_ in those cells [12,13,14,15]. Consequently, SODm could suppress tumor growth either in their own right or via enhancing radio- and chemotherapy, while protecting normal cells/tissues. Employing redox-active therapeutic strategies would thus constitute an alternative approach to cancer treatment and protection of normal tissues from the adverse effects of radio/chemotherapy [8,16,17].

Several classes of SODm have thus far been developed. The cationic Mn(III) *N*-alkyl- and *N*-alkoxyalkylpyridylporphyrins (MnPs) are among the most promising compounds. They are very stable and do not lose the metal active site under biological conditions. Due to their cationic nature, low molecular weight and fair lipophilicity, these compounds possess high bioavailability and distribute to different cellular organelles, mitochondria included. The SODm with the highest bioavailability bears long alky- and alkoxyalkyl groups, such as hexyl (MnTnHex-2-PyP^5+^) and butoxyethyl (MnTnBuOE-2-PyP^5+^) analogs [12].

**Figure 1:**
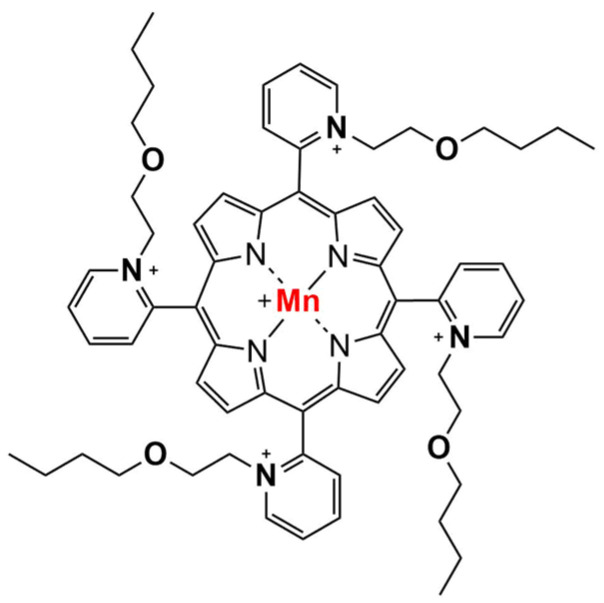
Chemical structure of MnTnBuOE-2-PyP^5+^ (chemical name: Mn(III) *meso*-tetrakis (*N*-n-butoxyethylpyridinium-2yl)porphyrin), described as MnBuOE throughout the text for simplicity.

The most recent third-generation Mn porphyrin is Mn(III) *meso*-tetrakis (*N*-n-butoxyethylpyridinium-2yl) porphyrin (MnTnBuOE-2-PyP^5+^, MnBuOE, BMX-001; Figure 1). This redox-active compound is an alkoxyalkyl analog of MnTnHex-2-PyP^5+^, which has already been extensively studied by our group [11,18,19]. Compared with the hexyl analog, an oxygen atom has been introduced into each of the four alkyl chains. This modification suppressed toxicity due to the disruption of surfactant properties while maintaining similar lipophilicity and redox properties as MnTnHex-2-PyP^5+^ [20]. Therefore, MnBuOE displays the optimal balance among toxicity, lipophilicity and bioavailability.

MnBuOE crosses the blood–brain barrier, which allows its use in the studies of brain-related pathologies [21,22]. It has been shown to improve memory and maintain dendritic length in mice exposed to chemotherapy [23] as well as to promote hippocampal neurogenesis after radiation [24]. MnBuOE has also been studied in head and neck cancer, rectal cancer and ovarian cancer [25,26,27]. Currently, MnBuOE is being evaluated in Phase I/II clinical trials as a radioprotector and chemosensitizer on glioma, head and neck cancer, multiple brain metastases and anal squamous cell carcinoma patients [12] (www.clinicaltrials.gov, accessed on 9 February 2023). Although MnBuOE has been studied in several types of cancer, there is a lack of information on the effect of this compound in lung cancer (LC). Worldwide, LC continues to be the main cause of cancer-related deaths for both genders combined [28,29], with non-small cell lung cancer (NSCLC) representing 85% of all LC cases [30]. NSCLC is frequently detected in advanced stages, when metastases have already developed, predominantly to the brain, liver, bones and adrenal glands [31]. The late detection of NSCLC cases, combined with the development of metastases, drastically decreases the survival rate to a very low percentage [32]. Cisplatin is the most frequently used chemotherapeutic drug [33]. The efficacy of cisplatin is due to its ability to react with the N-7 of purines in the nuclear DNA, creating intra- and inter-strand crosslinks and consequently blocking key cellular processes, which results in cell death [34,35]. One of the major limitations of cisplatin use is the development of chemoresistance, as well as its off-target toxicity, due to its indiscriminate attack on all rapidly dividing cells [36]. The most common adverse effects include nephrotoxicity, neurotoxicity, vomiting and ototoxicity [37].

Metastases are the main factor that contributes to the exceedingly low survival rate of NSCLC patients. Thus, our study intends to assess for the first time the therapeutic potential of MnBuOE as a single drug or combined with cisplatin on the development of metastases via evaluating cell viability and migration of NSCLC cancer cells (A549 and H1975 cell lines).

## 2. Materials and Methods

### 2.1. Chemicals

D-Glucose and HEPES were acquired from AppliChem (Darmstadt, Germany). Cisplatin, crystal violet (CV), extracellular matrix (ECM), penicillin–streptomycin (Pen/Strep) solution (10,000 units/mL of penicillin; 10 mg/mL of streptomycin) and sodium bicarbonate were obtained from Merck (Madrid, Spain). RPMI-1640 medium with L-glutamine was purchased from Biowest (Nuaillé, France). FBS and Trypsin (0.25%) were obtained from Gibco (Eugene, OR, USA). Sodium pyruvate was acquired from Lonza (Basel, Switzerland). CellTiter 96^®^ Aqueous MTS (3-(4,5-dimethylthiazol-2-yl)-5-(3-carboxymethoxyphenyl)-2-(4-sulphophenyl)-2H-Tetrazolium) was obtained from Promega (Madison, WI, USA). Acetic acid and ethanol absolute were acquired from Merck (Darmstadt, Germany).

MnBuOE was synthesized and characterized at Duke University School of Medicine. Stock solutions and respective dilutions were formulated in Mili-Q water. Cisplatin was dissolved in saline solution (0.9% NaCl) and its aliquoted solutions were kept at −20 °C. In all cell-based assays, controls were also included, in which cells were exposed to saline solution or Milli-Q water.

### 2.2. Cell Culture

The human NSCLC cell lines A549 and H975 were acquired from American Type Culture Collection (ATCC, Manassas, VA, USA). These two cell lines were cultured in monolayer in medium with L-glutamine supplemented with 2.5 g/L D-Glucose, 10 mM HEPES, 1.5 g/L sodium bicarbonate, 1 mM sodium pyruvate, 10% FBS and 1% Pen/Strep. This medium is defined as the complete cell culture medium. Cells were maintained at 37 °C and 5% CO_2_.

### 2.3. Crystal Violet Staining Assay

The cytotoxicity of MnBuOE per se in both NSCLC cells was evaluated by the CV staining assay as previously described [38]. Briefly, 3 × 10^3^ cells/well were seeded in 200 μL of complete medium in 96-well plates and incubated for 24 h at 37 °C under 5% CO_2_ atmosphere. After incubation, the culture medium was changed and cells were exposed to different MnBuOE concentrations (0.5–200 μM) for 72 h in order to assess a concentration–response profile. Cisplatin (50 μM) was used as a positive control for both cell lines. After the 72 h incubation period, PBS was used to wash the cells in order to remove non-adherent cells. The viable cells were fixed with ice-cooled EtOH and stained with 0.1% CV for 15 and 5 min, respectively. The excess of the dye was then removed with tap water and stained cells were resuspended with a solution of 96% EtOH/1% acetic acid. Absorbance was measured at 595 nm with a SPECTROstar OMEGA microplate reader (BMG Labtech, Offenburg, Germany). Absorbance values of the control cells were defined as 100% cell viability. Five independent experiments were performed and six replicates were used for each condition in each independent experiment. The Motic AE2000 Inverted Phase Contrast Microscope was used to perform image acquisition with a 40× objective.

### 2.4. MTS Reduction Assay

The MTS reduction assay was carried out in order to evaluate cell viability using the same conditions as mentioned for the CV assay and as previously described [39]. Briefly, cells were treated with a range of MnBuOE concentrations (0.5–100 μM) for a period of 72 h. After the treatment, the medium was removed and cells were washed with warm PBS. Cells were incubated with 100 μL of complete medium and 20 μL of MTS for 2 h. Cisplatin (50 μM) was used as a positive control. SPECTROstar OMEGA microplate reader was used to measure absorbance at 490 nm and 690 nm (reference wavelength). Absorbance values of control cells corresponded to 100% of cell viability. Four independent experiments were performed and three replicates were used for each condition in each independent experiment.

### 2.5. Cytotoxicity Assays of MnBuOE Combined with Cisplatin

The CV staining assay was performed in order to assess the cytotoxic effect of MnBuOE when combined with cisplatin in NSCLC cells. After the initial 24 h incubation, the culture medium was replaced and cells were co-treated with both compounds for 72 h. The concentrations of MnBuOE were the same for both cell lines (10 and 20 μM). Cisplatin concentrations used were 1 and 2 μM for A549 cells and 1 and 5 μM for H1975 cells. Four independent experiments were performed and six replicates were used for each condition in each independent experiment.

### 2.6. Intracellular ROS Evaluation

Cellular ROS levels analysis was performed using two different probes, dichloro-dihydro-fluorescein diacetate (DCF-DA) and dihydroethidium (DHE). DCF-DA is a ROS-dependent probe that is oxidized to dichlorofluorescein [40], while DHE is oxidized by superoxide to form hydroxyethidium. Even though DHE may react with other ROS and interfere with the fluorescence peak, the fluorescence intensity is mainly superoxide-dependent [41]. Briefly, A549 and H1975 cells were seeded at a density of approximately 12 × 10^4^ cells/well and incubated in a black 96-well plate with a clear bottom for 24 h at 37 °C. After seeding, both cells were exposed to MnBuOE (5 and 10 μM) alone or combined with cisplatin. A549 and H1975 cells were treated with 0.5 μM and 1 μM of cisplatin, respectively. Upon exposure, tert-butyl hydroperoxide (TBHP) (1 mM) and doxorubicin (10 μM) were added as positive controls of the DCF-DA and DHE probes, respectively, and incubated for 1 h at 37 °C. After this period, probes (10 μM) were added to each well and incubated for another hour at 37 °C. The Synergy HTX Multi-mode Microplate Reader (BioTek Instruments, Winooski, VT, USA) was used to measure fluorescence emission (DCF-DA and DHE: *λ_exc_* = 460 nm and *λ_em_* = 528 nm). Three independent experiments were conducted and five replicates were used for each condition in each independent experiment.

### 2.7. Cell Migration Assays

#### 2.7.1. Selection of MnBuOE and Cisplatin Concentrations for Migration/Invasion Assays

To establish the suitable non-cytotoxic concentrations for the migration and invasion assays, an MTS reduction assay was performed in both NSCLC cell lines, as previously described [39]. Briefly, 8 × 10^3^ cells/well were seeded in 96-well plates in complete culture medium for 24 h. After incubation, the complete medium was replaced by medium containing 2% FBS, and the A549 and H1975 cells were incubated with MnBuOE (5–20 μM) or cisplatin (0.25–1 μM) for 32 h. Three to four independent experiments were performed; three replicates were used for each condition in each independent experiment.

#### 2.7.2. In Vitro Wound-Healing Assay

An in vitro wound-healing assay was performed to evaluate the collective cell migration of both NSCLC cell lines, as previously described [38]. In short, A549 and H1975 cells were seeded in 24-well plates at ~8 × 10^4^ and 6.5 × 10^4^ cells/well, respectively, and incubated for 24 h in complete cell culture medium. Afterwards, the culture medium was removed and an injury was inflicted using a 200 μL sterile pipette tip, resulting in a scratch of about 0.6–0.8 mm wide. Warm PBS was used to wash the detached cells and cellular debris. The cells were then incubated in cultured medium with 2% FBS with the tested compounds. As such, H1975 cells were exposed to MnBuOE (5 μM) and/or cisplatin (1 μM) while A549 cells were exposed to MnBuOE (5 and 10 μM) and/or cisplatin (0.5 μM). The NSCLC cells were allowed to migrate up to 32 h. The Motic AE2000 Inverted Phase Contrast Microscope was used for image acquisition, with a 40× objective, and the Motic Images plus v2.0 software (Motic, Barcelona, Spain) was used to measure the injury width at 0, 8, 24 and 32 h for A549 cells and at 0, 20, 24 and 32 h for H1975 cells after the injury. The value of 0% of wound closure was considered at the initial timepoint of 0 h. The percentage of collective migration was then determined based on the initial distance of scratch. Two images of each scratch were taken at each time point for each condition (three replicates). Three different measures were made for each picture. Five to seven independent experiments were performed.

#### 2.7.3. Chemotaxis Migration Assay

A chemotactic migration assay was conducted to evaluate the single-cell migration capability of NSCLC cells when exposed to the compounds, based on a protocol already described [11,38]. This assay was performed in 24-well plates with transwell inserts with transparent polyethylene terephthalate (PET) membranes containing 8 μm pores (BD Falcon, Bedford, MA, USA). A549 and H1975 cells were seeded in 24-well plates at approximately 2 × 10^4^ and 5 × 10^4^ cells/well, respectively, in 2% FBS medium on top of the transwell insert; complete culture medium was placed in the lower chamber, functioning as a chemoattractant. After seeding, H1975 cells were exposed to MnBuOE (5 μM) and/or cisplatin (1 μM) and A549 cells were subjected to MnBuOE (5 and 10 μM) and/or cisplatin (0.5 μM). The compounds were added to both compartments, and H1975 and A549 cells were incubated for 20 h and 24 h, respectively. After this period, non-migrating cells were gently removed from the upper chamber with a cotton swab. Migrating cells at the bottom of the membrane were then fixed with a volume of 600 μL of 96% ethanol (for 10 min) and stained with 0.1% CV (for 15 min). The inserts were washed with tap water and dried out for 24 h. Five images were taken for each condition, using a Motic S6 (Motic, Barcelona, Spain) placed on a Motic AE2000 Inverted Phase Contrast Microscope with an amplification of 100×. The software Motic Images plus v3.0 (Motic, Barcelona, Spain) was used to count the stained cells from each picture. The number of cells was expressed as percentage of control and three independent experiments were performed.

### 2.8. Chemoinvasion

The invasive capability of NSCLC cells was analyzed through a chemoinvasion assay, based on a protocol reported prior [38]. This procedure is very similar to the one previously detailed in the chemotaxis assay. The main difference is the addition of 50 μL of extracellular matrix (ECM) gel (1:25 dilution in serum-free medium). The addition of this coating step mimics the structure of the extracellular matrix, allowing the observation of an invasion-like process, similar to in vivo conditions. The seeding density was approximately 1.4 × 10^5^ and 2 × 10^4^ cells/well for A549 and H1975 cells, respectively. The concentrations used and the analysis of results were conducted similarly to the abovementioned chemotactic migration assay. Three independent experiments were performed.

### 2.9. Gene Expression

The expression of several genes related with migration and invasion (*MMP2, MMP9, CDH1, VIM, EGFR* and *VEGFA*) was measured using quantitative reverse transcription PCR (RT-qPCR), as previously described but with some alteration [19]. A549 cells and H1975 cells were seeded in a 6-well plate at a density of approximately 1.9 × 10^5^ cells/well and 2.25 × 10^5^ cells/well, respectively, and incubated for 24 h at 37 °C. After seeding, cells were exposed to MnBuOE alone or combined with cisplatin, using the same concentrations as in migration and invasion assays. Upon exposure, total RNA was isolated using TRIzol^TM^ Reagent (Invitrogen, Waltham, MA, USA). After quantification, cDNA was synthesized using a commercially available kit (NZYTech, Lisbon, Portugal). The qPCR was performed with PowerUp™ SYBR^®^ Green Master Mix (Applied Biosystems^®^/Life Technologies, Austin, TX, USA), according to the manufacturer’s instructions. A final reaction volume of 5 μL, with 0.333 μM of forward and reverse primers (Table 1) and 1 μL of template cDNA, was used and the reaction was conducted in the QuantStudio™ 7 Flex Real-Time PCR System (Applied Biosystems™, Waltham, MA, USA, EUA) according to Cipriano et al. [42]. A dissociation stage was inserted to determine the melting temperature in all runs, as a specificity and quality measurement. Gene expression was quantified through the comparative Ct method (2^−ΔΔCt^) and normalized to a reference gene (*β*-actin).

### 2.10. Statistical Analysis

The normality and the homogeneity of the variances of continuous variables were first assessed. Afterwards, the differences in mean values of the results were evaluated by two-tailed Student’s *t*-test or by one-way analysis of variance (ANOVA) followed by Tukey’s multiple comparison test. The SPSS statistical package (version 25; SPSS Inc., Chicago, IL, USA) was used to perform all statistical analyses.

## 3. Results

### 3.1. Effect of MnBuOE on Cell Viability in NSCLC Cells

**Figure 2:**
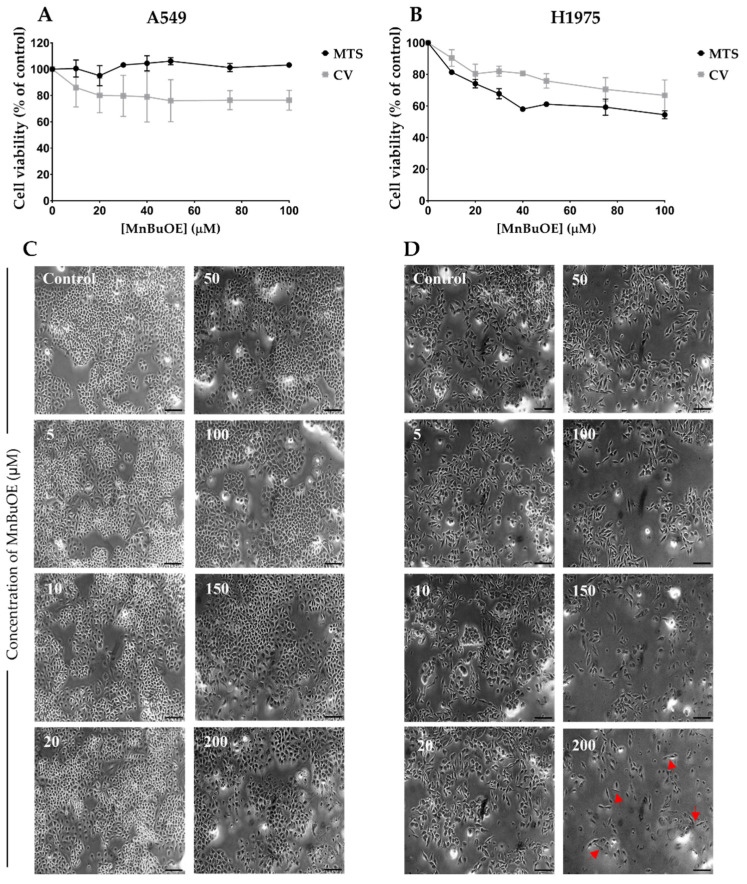
Cytotoxic effects of MnBuOE (5–200 μM) in A549 and H1975 cells. Upon exposure to MnBuOE for 72 h, the cell viability was assessed by CV and MTS assays in (**A**) A549 and (**B**) H1975 cells. Values represent mean ± SD (*n* = 3–6) and are expressed as percentages of the vehicle-treated control cells. (**C**) A549 cell morphology upon exposure to MnBuOE. (**D**) H1975 cell morphology upon exposure to MnBuOE. Red arrows point to changes in morphology. Scale bar = 100 μm.

The effect of MnBuOE was assessed in two NSCLC cell lines with two complementary methodologies, CV staining and MTS reduction assays. Both assays demonstrated that high concentrations of MnBuOE were required to induce cytotoxic effects in both cell lines. No cytotoxic effects were observed for A549 cells, up to 100 μM, in the MTS assay. A modest decrease in cell viability of approximately 25% was observed with the CV assay for the highest concentration tested (Figure 2A). In the case of H1975 cells, both methodologies pointed to a higher reduction in cell viability. In the MTS assay, the maximum concentration tested induced cytotoxicity of approximately 45%, while in the CV staining assay the maximum concentration tested led to a reduction in cell viability of approximately 33% (Figure 2B). Although the 100 μM concentration could already be considered high, we further tested the MnP in both cell lines using the CV assay up to 200 μM. This concentration only led to an additional small decrease in cell viability by approximately 5% and 15% in A549 and H1975 cells, respectively. Overall, these results demonstrate a low cytotoxic pattern displayed by MnBuOE.

H1975 cells appear to be more sensitive to MnBuOE than A549 cells. Regarding cell morphology, A549 cells maintained their features within the range of MnBuOE concentrations studied (Figure 2C), while in H1975 cells it is possible to observe a stretching of the cell at the highest concentration tested, as indicated by the red arrows in Figure 2D, resembling a fibroblast morphology. Based on such results, the 10 and 20 μM MnBuOE concentrations were chosen for the combinatory assays.

### 3.2. Impact of MnBuOE Combined with Cisplatin on the Viability of NSCLC Cells

**Figure 3:**
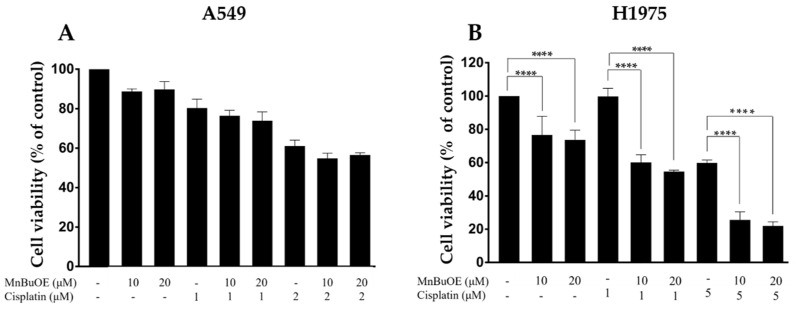
Cytotoxic effect of MnBuOE combined with cisplatin in A549 and H1975 cells. (**A**) A549 cells were treated with MnBuOE (10 and 20 μM) and cisplatin (1 and 2 μM) while (**B**) H1975 cells were incubated with MnBuOE (10 and 20 μM) and cisplatin (1 and 5 μM) for 72 h and assessed by CV assay. Values represent mean ± SD (*n* = 2–4) and are expressed as percentages relative to control cells. **** *p* < 0.0001 (one-way ANOVA with Tukey’s multiple comparisons test) when compared with control and cisplatin-treated cells.

To assess if MnBuOE could potentiate the cytotoxicity of cisplatin, both drugs were simultaneously added to A549 and H1975 cells and the effects were evaluated using the CV staining assay. In A549 cells, the co-incubation of MnBuOE with cisplatin did not lead to a significant effect compared with MnBuOE alone. A marginal decrease in terms of cell viability in co-treated cells was observed (Figure 3A).

In contrast, the combination of MnBuOE with cisplatin significantly reduced the viability of H1975 cells under all conditions tested (*p* < 0.0001) (Figure 3B). The lowest cisplatin concentration (1 μM) did not display any cytotoxicity, whereas the highest concentration (5 μM) induced a cytotoxic effect of approximately 40%. Yet, when cisplatin was combined with MnBuOE, its cytotoxicity was significantly enhanced. The combination of 1 μM cisplatin with 10 μM MnBuOE impaired cell viability by 39.6% (*p* < 0.0001). When 1 μM cisplatin was combined with 20 μM of MnBuOE, cells suffered a reduction in cell viability of 45.2% (*p* < 0.0001). The combination of the same MnP concentrations (10 and 20 μM) with 5 μM cisplatin induced a reduction in cell viability of 34.3% (*p* < 0.0001) and 37.9% (*p* < 0.0001), respectively. The decreases in cell viability of the combinatory conditions were calculated in relation to the percentage of cisplatin alone. So, even though there was no statistically significant effect of MnBuOE as a single drug on A549 cells, there was an impressive reduction in viability in H1975 cells when MnBuOE was combined with cisplatin.

### 3.3. MnBuOE per se and Combined with Cisplatin Increases ROS Levels

**Figure 4:**
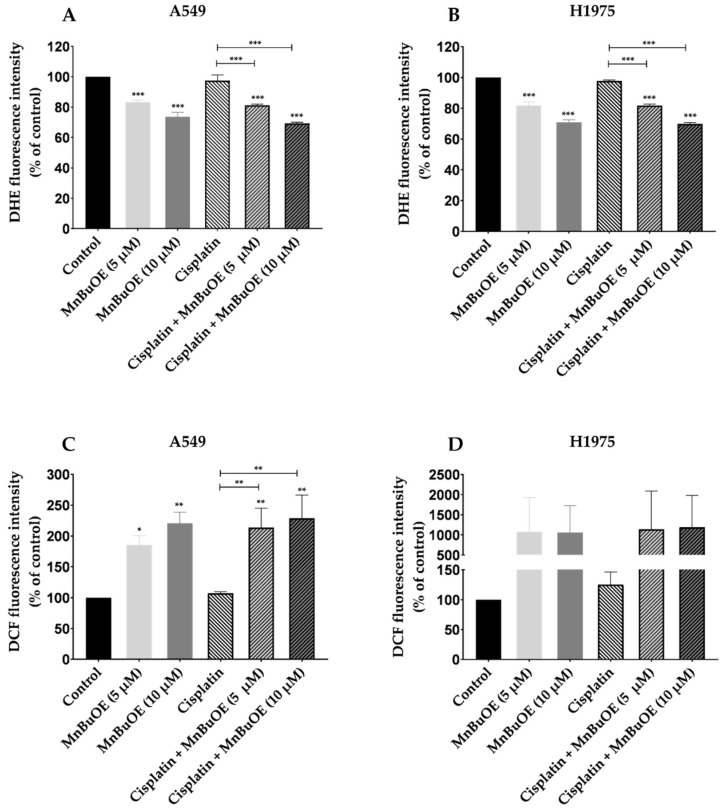
Intracellular ROS levels of NSCLC cells exposed to MnBuOE alone or combined with cisplatin. ROS levels were measured for 24 h using two fluorescent probes: a DHE probe on (**A**) A549 cells and (**B**) H1975 cells and a DCF-DA probe on (**C**) A549 cells and (**D**) H1975 cells. Values represent the mean ± SD (*n* = 3–4) and are expressed as percentages relative to control cells. * *p* < 0.05, ** *p* < 0.01 and *** *p* < 0.001 (one-way ANOVA with Tukey´s multiple comparisons test) when compared with control and cisplatin-treated cells.

The levels of intracellular ROS in NSCLC cells exposed to MnBuOE alone or combined with cisplatin were assessed by fluorimetry using DHE and DCF-DA fluorescence probes (Figure 4). Regarding the DHE probe, similar results were obtained in both cell lines. Fluorescence intensity significantly decreased upon treatment with MnBuOE alone or combined with cisplatin in a concentration-dependent manner (*p* < 0.001) (Figure 4A,B). In cells treated with MnBuOE alone, a reduction in intracellular ROS levels of approximately 17% (*p* < 0.001) and 27% (*p* < 0.001) was detected at the concentrations of 5 and 10 μM, respectively. When combined with cisplatin, MnBuOE also significantly reduced fluorescence intensity compared with controls or cisplatin-treated cells (*p* < 0.001).

In the case of the DCF-DA probe, MnBuOE was able to significantly increase intracellular ROS levels when compared with non-treated control cells, either alone or combined with cisplatin, with this increase being concentration-dependent in A549 cells (Figure 4C). The lowest concentration of MnBuOE (5 μM) increased fluorescence intensity by approximately 85% (*p* < 0.05), while the highest concentration of MnBuOE (10 μM) raised ROS levels by 120% (*p* < 0.01). When combined with cisplatin, MnBuOE increased fluorescence intensity by approximately 114% (*p* < 0.01) and 129% (*p* < 0.01) at the concentrations of 5 and 10 μM, respectively. In H1975 cells, treatment with MnBuOE per se or combined with cisplatin did not lead to a significant increase in ROS levels when compared with non-treated cells (Figure 4D). Nevertheless, it is possible to observe a tendency in the increase in fluorescence intensity when cells were treated with MnBuOE alone or combined with cisplatin.

### 3.4. MnBuOE per se and Combined with Cisplatin Reduces Collective Migration of NSCLC Cells

To assess the effect of cytotoxic compounds on migration and invasion, it is necessary to use non-cytotoxic concentrations to confirm that the results obtained are due to an impairment in cell migration and not to a reduction in cell viability. Cell viability assays using culture medium with 2% FBS were performed to ensure that there was no decrease in cell viability under the conditions of the cell migration assays.

**Figure 5:**
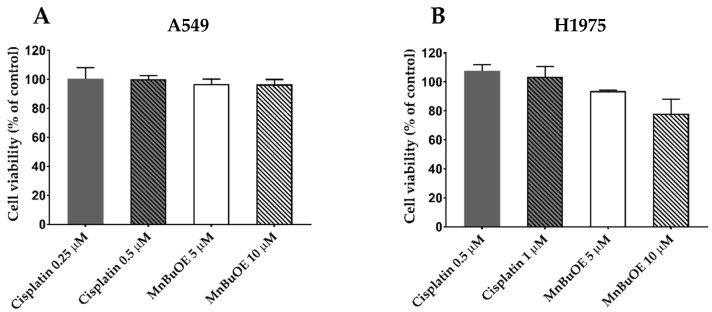
Viability of NSCLC cells when exposed to low concentrations of cisplatin or MnBuOE in a 2% FBS cell culture medium. (**A**) Effect of cisplatin (0.25 and 0.5 μM) and MnBuOE (5 and 10 μM) on the viability of A549 cells. (**B**) Effect of cisplatin (0.5 and 1 μM) and MnBuOE (5 and 10 μM) on the viability of H1975 cells. The MTS reduction assay was performed as a cell viability assessment. Values represent mean ± SD (*n* = 3) and are expressed as percentages relative to control cells.

A549 cells were treated with 0.25 and 0.5 μM cisplatin, whereas H1975 cells were incubated with 0.5 and 1 μM cisplatin. Both cell lines were treated with the same MnBuOE concentrations of 5 and 10 μM. All experiments were performed for 32 h and the cytotoxic effects were assessed by MTS assay. In A549 cells, both cisplatin concentrations displayed a cell viability of 100%; so, the concentration of 0.5 μM was selected for the following experiments. In the case of MnBuOE, none of the concentrations tested led to a reduction in cell viability; thus, both concentrations were selected for the migration and invasion assays (Figure 5A).

With H1975 cells, both cisplatin concentrations did not lead to a loss in cell viability. MnBuOE at 5 μM decreased cell viability by less than 10%. Since the concentration of 10 μM MnBuOE was already cytotoxic, we chose the non-cytotoxic concentrations of 1 μM cisplatin and 5 μM MnBuOE (Figure 5B).

**Figure 6:**
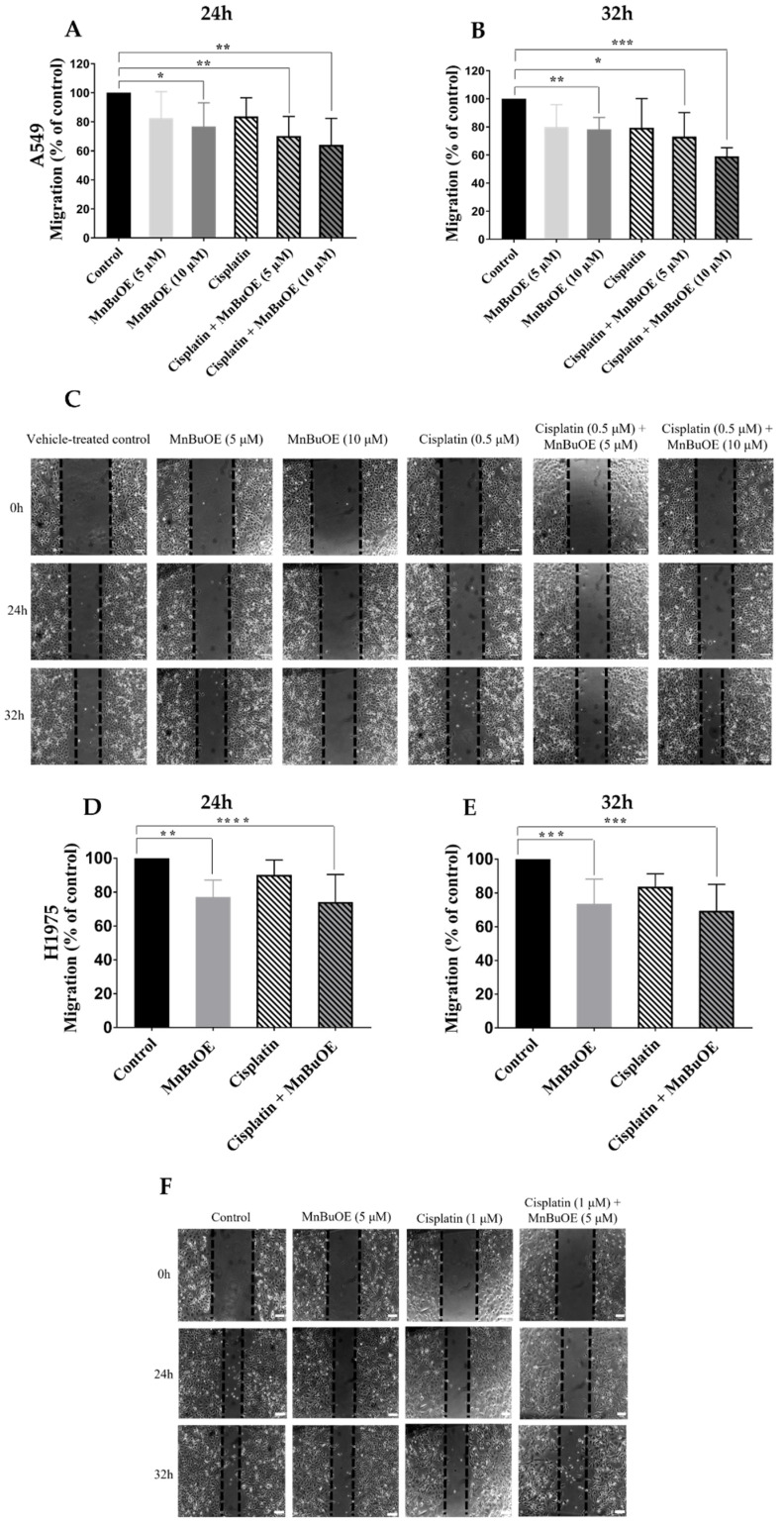
The effect of MnBuOE alone or combined with cisplatin on the collective migration of NSCLC cells. Cell migration was evaluated by the wound-healing assay using ((**A**), 24 h; (**B**), 32 h) MnBuOE (5 and 10 μM) and cisplatin (0.5 μM) in A549 cells and ((**D**), 24 h; (**E**), 32 h) using MnBuOE (5 μM) and cisplatin (1 μM) in H1975 cells. Representative microscopy images of the wound-healing assay of (**C**) A549 and (**F**) H1975 cells are demonstrated. Values for the wound-healing assay represent mean ± SD (*n* = 5–6) and are expressed as percentages of wound closure, calculated relative to the initial wound width. Statistical analysis was performed for each timepoint, comparing each condition with the control cells; * *p* < 0.05, ** *p* < 0.01, *** *p* < 0.001 and **** *p* < 0.0001 (Student´s *t*-test). Scale bar = 100 μm.

In A549 cells, MnBuOE significantly reduced collective cell migration both alone and when combined with cisplatin at 24 h and 32 h (Figure 6A–C). At the highest concentration tested (10 μM), MnBuOE was able to reduce migration by approximately 24% at 24 h (*p* < 0.05) and 32 h (*p* < 0.01). The lowest concentration tested was able to decrease migration by approximately 20%, albeit this difference was not statistically significant. However, when MnBuOE was combined with cisplatin, a higher reduction in cell motility was demonstrated. When cisplatin was combined with 5 and 10 μM MnBuOE, there was a decrease in migration of ~30% (*p* < 0.01) and 36% (*p* < 0.01) at 24 h, respectively, and 29% (*p* < 0.05) and 41% (*p* < 0.001) at 32 h, respectively. The data suggest that the effect of MnBuOE is concentration-dependent.

MnBuOE also reduced cell motility both alone and combined with cisplatin in H1975 cells. MnBuOE alone impaired collective migration by approximately 13% (*p* < 0.001) and 18% (*p* < 0.01) at 24 h and 32 h, respectively (Figure 6D–F). Similarly to A549 cells, the best condition tested was the MnP/cisplatin combination. Such treatment was able to reduce migration by 30% (*p* < 0.001) and 37% (*p* < 0.001) at 24 h and 32 h, respectively. Overall, MnBuOE appeared to have a stronger effect on the migration than on the cell viability. Thus, further assays related to cell migration were performed to better understand the impact of this MnP.

### 3.5. MnBuOE Alone and in Combination with Cisplatin Reduces Chemotactic Cell Migration

Individual cell migration is a crucial step in the development of metastases to peripheral organs. Single-cell migration happens in the early stages of invasion in the metastatic process [50]. For this reason, the impact of MnBuOE alone or combined with cisplatin on chemotactic cell migration was evaluated by a transwell migration assay.

**Figure 7:**
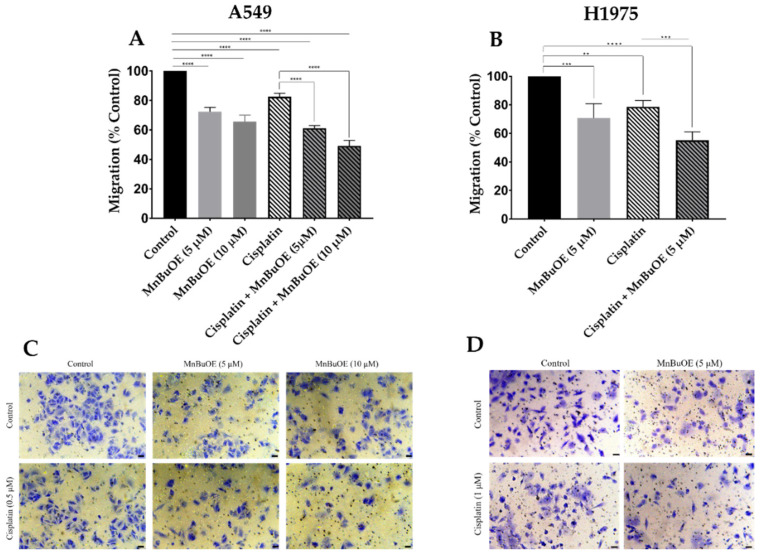
The effect of MnBuOE on chemotactic migration of NSCLC cells exposed to cisplatin. Chemotactic cell migration was measured using a transwell migration assay in (**A**) A549 and (**B**) H1975 cells. Representative microscopy images of the transwell migration assay are shown, where migrated (**C**) A549 and (**D**) H1975 cells are stained with crystal violet. Values represent mean ± SD (*n* = 3) and are expressed as percentages of control cells. ** *p* < 0.01, *** *p* < 0.001 and **** *p* < 0.0001 (one-way ANOVA with Tukey´s multiple comparisons test) when compared with control and cisplatin-treated cells. Scale bar = 150 μm.

In both NSCLC cell lines, MnBuOE significantly reduced cell migration, either alone or combined with cisplatin. The concentrations used were the same as in the evaluation of the collective cell migration. In A549 cells, MnBuOE as a single drug was able to significantly reduce this type of migration in a concentration-dependent manner (Figure 7A). The 5 and 10 μM MnBuOE reduced the extent of cell migration by 28% (*p* < 0.0001) and 35% (*p* < 0.0001), respectively. Although cisplatin alone significantly decreased individual migration (*p* < 0.0001), MnBuOE combined with cisplatin exhibited the largest reduction in chemotactic cell migration. The reduction caused by MnBuOE/cisplatin combination also occurred in a concentration-dependent manner, i.e., cisplatin combined with 5 μM and 10 μM MnBuOE impaired cell migration by 39% (*p* < 0.0001) and 51% (*p* < 0.0001), respectively.

Similarly to A549 cells, MnBuOE alone reduced individual migration of H1975 cells by approximately 30% (*p* < 0.01) (Figure 7B). Cisplatin alone also significantly reduced migration (*p* < 0.01). Yet, when combined, the largest reduction in individual cell migration of ~45% was demonstrated (*p* < 0.0001). The decrease was also significant when the impact of MnBuOE/cisplatin was compared with cisplatin-only-treated cells (*p* < 0.001). Representative images of the chemotactic migration assay are represented in Figure 7C,D (A549 and H1975 cells, respectively).

### 3.6. MnBuOE per se and Combined with Cisplatin Decreases Chemoinvasion of NSCLC Cells

The transwell chemoinvasion assay was performed similarly to the transwell chemotactic assay but with the addition of ECM gel to better mimic the passage through basement membranes.

**Figure 8:**
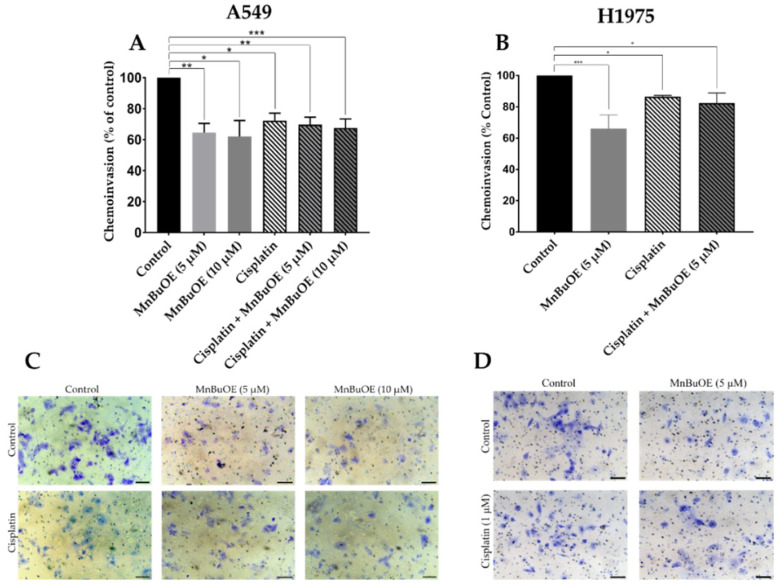
Effect of MnBuOE alone or combined with cisplatin on chemoinvasion of NSCLC cells. Transwell chemoinvasion was evaluated (**A**) for a 24 h period on A549 cells and (**B**) for a 20 h period on H1975 cells. Representative microscopy images of (**C**) A549 and (**D**) H1975 invading cells stained with crystal violet are shown. Values represent the mean ± SD (*n* = 3) and are expressed as percentages relative to control cells. * *p* < 0.05, ** *p* < 0.01 and *** *p* < 0.001 (one-way ANOVA with Tukey´s multiple comparisons test) when compared with control. Scale bar = 150 μm.

Unlike the results observed in collective and individual cell migration, in the cell invasion assay, MnBuOE alone reduced cell invasion to the largest extent on both cell lines. In A549 cells, 5 μM and 10 μM MnBuOE alone decreased cell invasion by approximately 35% (*p* < 0.01) and 38% (*p* < 0.05), respectively (Figure 8A). When combined with cisplatin, 5 μM MnBuOE reduced invasion by 30% (*p* < 0.01) and 10 μM MnBuOE by 32% (*p* < 0.001). In this cell line, no correlation with concentration was observed. In H1975 cells, MnBuOE alone reduced invasion by ~34% (*p* < 0.001) (Figure 8B). When combined with cisplatin, MnBuOE reduced invasion by ~18% (*p* < 0.05). Representative images of the chemoinvasion assay are represented in Figure 8C,D.

### 3.7. Effect of MnBuOE on the Expression of a Panel of Cell Migration-Related Genes in NSCLC Cells

**Figure 9:**
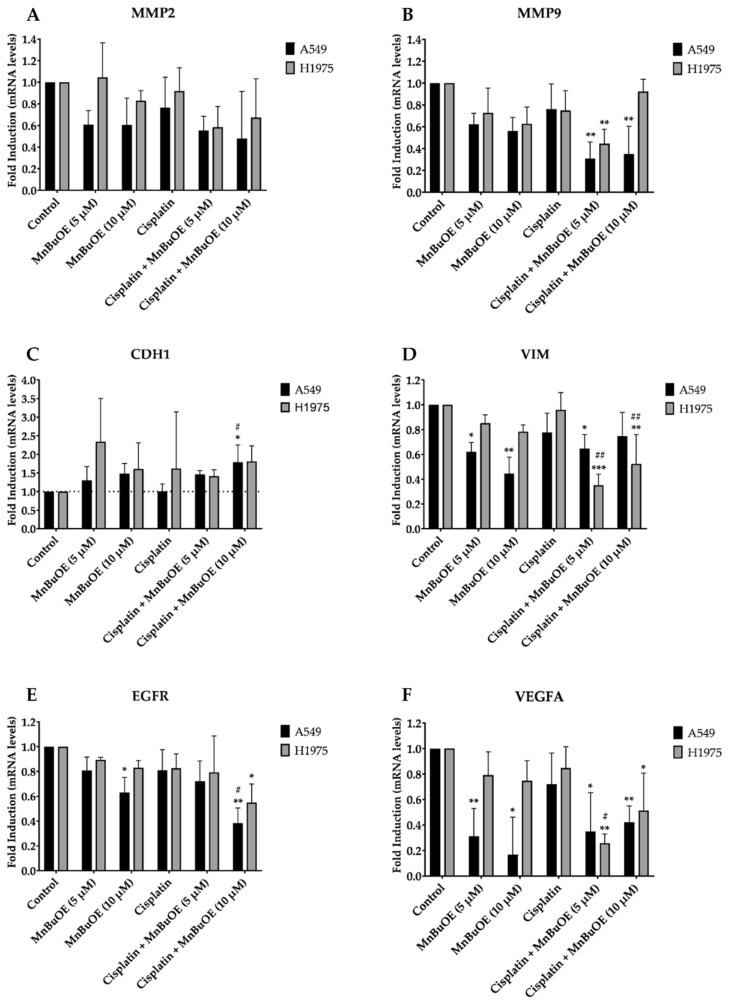
MnBuOE alone or combined with cisplatin altered gene expression of several migration- and invasion-related genes. MnBuOE downregulated (**A**) *MMP2*, (**B**) *MMP9*, (**D**) *VIM*, (**E**) *EGFR* and (**F**) *VEGFA* in NSCLC cell lines and upregulated (**C**) *CDH1*. Values represent mean ± SD (*n* = 3) and are normalized to the reference gene β-actin. * *p* < 0.05, ** *p* < 0.01 and *** *p* < 0.001 (one-way ANOVA with Tukey´s multiple comparisons test) when compared with control. ^#^ *p* < 0.05 and ^##^ *p* < 0.01 when compared with cisplatin-treated cells.

To better understand the effect of MnBuOE on cell migration and invasion, we evaluated the expression of several genes related with these cellular processes in NSCLC cells exposed to MnBuOE alone or combined with cisplatin (Figure 9). Overall, A549 cells displayed a pronounced downregulation of *MMP2, MMP9, VIM, EGFR* and *VEGFA* when exposed to the MnP alone or combined with cisplatin. In the case of H1975 cells, the level of gene expression dysregulation was less evident and not significant in many cases. Nevertheless, we observed a general downregulation of these genes in both cell lines and an overexpression of *CDH1* when compared with non-treated cells.

MMP2 and MMP9 (two matrix metalloproteinases) can degrade collagen, allowing cells to initiate an epithelial-to-mesenchymal transition (EMT) and to invade the surrounding tissues. NSCLC cells exposed to MnBuOE alone or combined with cisplatin present a downregulation of these two genes (Figure 9A,B). E-cadherin (CDH1) plays a crucial role in cellular adhesion; however, it is usually downregulated in several types of cancer, facilitating invasive growth [51]. In both cell lines, MnBuOE alone or combined with cisplatin caused an upregulation of *CDH1* (Figure 9C). Vimentin (VIM) is a protein present in cellular filaments that promotes migration of cancer cells. However, cells treated with MnBuOE and cisplatin + MnBuOE expressed lower levels of *VIM* than non-treated cells (Figure 9D). Finally, we observed a downregulation of two growth factors involved in cellular proliferation and migration, i.e., epidermal growth factor receptor (*EGFR*) and vascular endothelial growth factor *α* (*VEGFA*). This effect was more pronounced in A549 cells (Figure 9E,F).

## 4. Discussion

The standard treatment of NSCLC requires improvement in order to increase the efficacy and tolerability of chemotherapy, i.e., decreasing its off-target toxicity and overcoming the resistance acquired by cancer cells. For that to be achieved, it is urgent to find new drugs that could be used alone or in combination with standard-of-care chemotherapy. As noted, cancer cells generally possess higher levels of reactive species due to either downregulation of antioxidant enzymes or their inactivation when compared with healthy cells. The mimics of the superoxide dismutase enzyme family, most so cationic Mn porphyrins, were created as a promising therapeutic strategy to induce cancer cell death while protecting normal cells [5,7,8]. Two of those compounds, i.e., MnBuOE (BMX-001) and MnTE-2-PyP^5+^ (BMX-010) are currently in several Phase I/II clinical trials (see Section 1). Numerous studies have demonstrated that MnBuOE can increase the efficacy of chemo and radiotherapy. Ashcraft et al. [25] demonstrated that MnBuOE broadened the therapeutic window of radiotherapy in head and neck cancer by decreasing the dose of radiation necessary to control the tumor and by increasing the resistance of normal tissues to the injury caused by radiation. Also, this compound has been shown to enhance the chemotherapy treatment efficacy in rectal and anal cancers [26] as well as in breast cancer and glioma [12]. Recently, Chaiswing et al. performed an in vitro study where MnBuOE sensitized carboplatin-resistant ovarian cancer cells when exposed to concentration levels as low as 50 nM while protecting healthy ovarian cells from chemo-induced injuries [27]. Besides enhancing the effects of radio- and chemotherapy, this MnP also protects normal tissue from these treatments (review in [12,14,20]) but does not protect cancer cells. Such a differential effect enabled the progress of MnBuOE into clinical trials in Oncology [12].

Although MnPs have already been tested in several types of cancer using different cellular and animal models, there is still a lack of information regarding the cytotoxic effect of MnPs in lung cancer. We have previously demonstrated the high cytotoxicity of a lipophilic hexyl compound, MnTnHex-2-PyP^5+^, on NSCLC [19]. The addition of oxygen atoms into the hexyl chains reduces its surfactant properties and consequently originates a less toxic and lipophilic compound, MnBuOE [12,20]. Herein, our goal was to assess the impact of this clinically developed analog, as a single drug or combined with cisplatin, on the viability and migration of cancer cells aiming at the prevention and reduction of metastases.

Our in vitro work was carried out on two representative human NSCLC cell lines. The H1975 cell line originated from a non-smoker female with lung adenocarcinoma, the most common subtype of NSCLC [30], being a highly invasive cell line [52]. This cell line has a mutation that confers its resistance to EGFR inhibitors [53]. The A549 cell line is one of the most used cell lines in NSCLC studies, allowing us to compare our results with those of other studies. It possesses a K-RAS mutation [54] and is sensitive to cisplatin [55].

We first evaluated the cytotoxic effects of MnBuOE per se in both cell lines using two complementary methodologies. While H1975 cells were more sensitive to MnBuOE, only moderate cytotoxicity to A549 cells was displayed. Yulyana et al. demonstrated the impact of MnBuOE on glioblastoma. A human glioma cell line (ΔGli36) and an immortalized normal human astrocytes cell line (iNHA) were exposed to high concentrations of MnBuOE (up to 500 μM). The ΔGli36 cells suffered a decrease in cell viability of approximately 30%, similar to the cytotoxic profile observed in A549 cells [56].

Previously, we reported the different sensitivities of H1975 and A549 cell lines towards cisplatin [19]. This study demonstrates that MnBuOE enhances the cytotoxicity of cisplatin in H1975 cells, which agrees well with reports on radio- and chemosensitizing properties of the whole class of cationic Mn porphyrins [12,14,20]. The lack of a significant effect on the A549 cell line might be due to the lower sensitivity of A549 cells when exposed to MnBuOE alone. Important to note is the strong chemosensitizing effect of MnBuOE on H1975 cells, a cell line more resistant to cisplatin than A549 cells.

The results obtained with the two fluorescent probes corroborate the mode of action suggested for these redox-active compounds. In the case of the DHE probe, MnBuOE significantly decreased intracellular ROS levels in both cell lines, with this decrease being concentration dependent. The DHE probe mostly reacts with O_2_^•−^, although its reaction with other reactive species may affect the fluorescence peak [57,58]. SODm dismute O_2_^•−^ into H_2_O_2_ and oxygen; therefore, the decrease in the fluorescence intensity of DHE, when cells are exposed to MnBuOE alone or combined with cisplatin, corroborates the suggested mode of action. Fernandes et al. assessed the intracellular ROS levels in V79 cells exposed to MnTnHex-2-PyP^5+^, an analog of MnBuOE, using the same probe. A significant decrease in fluorescence intensity in cells treated with MnTnHex-2-PyP^5+^ was reported [57]. Regarding the DCF-DA probe, that detects several ROS, we observed that MnBuOE increased intracellular ROS levels in both NSCLC cell lines, and this increase was concentration-dependent in A549 cells. Although not significant, the magnitude of ROS increases was much more noticeable in H1975. These results suggest a lower antioxidant capability of H1975 cells, which may explain the sensitivity of these cells when exposed to MnBuOE alone or combined with cisplatin in comparison with A549 cells. These results also indicate that cisplatin does not influence the production of ROS when combined with MnBuOE, since the percentage of fluorescence intensity is very similar to that of MnBuOE alone in both probes. In renal cancer cells, MnTnHex-2-PyP^5+^ also increased intracellular ROS levels [18]. Therefore, our results are in agreement with the literature.

Our study further demonstrated that MnBuOE alone or combined with chemotherapy was able to significantly reduce migration and invasion in both cell lines. With regards to collective and individual migration, the most effective condition was the co-treatment with MnBuOE and cisplatin. Finally, MnBuOE alone exhibited higher invasion reduction in both cell lines than when combined with cisplatin. Although there is a lack of knowledge on the effect of MnBuOE on migration, several studies have been conducted with its analog MnTnHex-2-PyP^5+^. In renal cancer, this MnP significantly reduced chemotactic migration [18], and when combined with doxorubicin, it decreased cell motility and chemotactic migration in breast cancer cells [11]. The differential response of A549 and H1975 cells with regards to cytotoxicity and migration/invasion may be related to their different redox profiles and differential interference with metastatic pathways. In our previous work, we analyzed the gene expression of the main H_2_O_2_ detoxifying enzymes and observed that, despite low levels of catalase in both cell lines, A549 cells had lower levels of peroxiredoxin 2 (*PRDX2*) and higher levels of peroxiredoxin 1 (*PRDX1*) than H1975 cells [19]. This difference could in part be explained by differences observed between these cell lines with regards to MnBuOE/cisplatin treatments.

In order to better understand the effect of MnBuOE on migration and invasion in these cells, we analyzed this effect from a mechanistic perspective; for this purpose, the expression of several genes that have been identified as key players in migration and invasion was evaluated. The downregulation of *MMP2* and *MMP9*, and the upregulation of *CDH1*, on both cell lines are in agreement with the results observed in the chemoinvasion assay. Since MMPs are involved in the EMT process and *CDH1* is usually downregulated in cancer cells, the downregulation of *MMPs* and upregulation of *CDH1* in cells exposed to MnBuOE contribute to the reduction in invasion. Also, the downregulation of *VIM, EGFR* and *VEGFA* are in alignment with the results observed in the collective and chemotactic migration since these genes are involved in migration processes. It is worth noting that in A549 cells, the downregulation of these genes was more evident than in H1975 cells, which may explain the higher effect of MnBuOE in reducing migration in this cell line. Although MnBuOE and its analogs have never been studied in the context of NSCLC, some reports demonstrated their anti-metastatic potential in other types of cancer. In colorectal cancer, Yang et al. showed that the ethyl analog, MnTE-2-PyP^5+^, reduced the expression of mesenchymal markers in SW480 cells and suppressed the expression of MMP2 and MMP9 [59]. In a mouse subcutaneous xenograft glioma model, MnBuOE downregulated proteins involved in metastases [60]. Jointly, such studies further strengthen the therapeutic potential of the whole class of cationic Mn(III) *N*-alkyl- and *N*-alkoxyalkylpyridylporphyrins in suppressing metastases in NSCLC cell lines.

## 5. Conclusions

In summary, this work suggests that MnBuOE is a promising drug candidate to prevent and/or reduce the development of metastases in NSCLC, one of the main drivers responsible for the low survival rate of LC.

## Figures and Tables

**Table 1:** Primers used for qRT-PCR for characterization of NSCLC cells exposed to MnBuOE alone or combined with cisplatin.

Gene	Forward Primer (5′-3′)	Reverse Primer (5′-3′)	Reference
*β-ACTIN*	CATGTACGTTGCTATCCAGGC	CTCCTTAATGTCACGCACGAT	[43]
*MMP2*	TGACTTTCTTGGATCGGGTCG	AAGCACCACATCAGATGACTG	[44]
*MMP9*	TGTACCGCTATGGTTACACTCG	GGCAGGGACAGTTGCTTCT	[45]
*CDH1*	CTGAGAACGAGGCTAACG	GTCCACCATCATCATTCAATAT	[46]
*VIM*	AGTCCACTGAGTACCGGAGAC	CATTTCACGCATCTGGCGTTC	[47]
*EGFR*	AGGCACGAGTAACAAGCTCAC	ATGAGGACATAACCAGCCACC	[48]
*VEGFA*	AGGGCAGAATCATCACGAAGT	AGGGTCTCGATTGGATGGCA	[49]

## Data Availability

The data that support the findings of this study are available from the corresponding author upon reasonable request.
